# Reliability and validity of the Microsoft Kinect for evaluating static foot posture

**DOI:** 10.1186/1757-1146-6-14

**Published:** 2013-04-08

**Authors:** Benjamin F Mentiplay, Ross A Clark, Alexandra Mullins, Adam L Bryant, Simon Bartold, Kade Paterson

**Affiliations:** 1 School of Exercise Science, Faculty of Health Sciences, Australian Catholic University, Melbourne, Australia; 2 Department of Physiotherapy, Faculty of Medicine, Dentistry and Health Sciences, The University of Melbourne, Melbourne, Australia

**Keywords:** Foot morphology, Injury screening, Clinical assessment, Lower limb, Biomechanics, Anthropometry

## Abstract

**Background:**

The evaluation of foot posture in a clinical setting is useful to screen for potential injury, however disagreement remains as to which method has the greatest clinical utility. An inexpensive and widely available imaging system, the Microsoft Kinect™, may possess the characteristics to objectively evaluate static foot posture in a clinical setting with high accuracy. The aim of this study was to assess the intra-rater reliability and validity of this system for assessing static foot posture.

**Methods:**

Three measures were used to assess static foot posture; traditional visual observation using the Foot Posture Index (FPI), a 3D motion analysis (3DMA) system and software designed to collect and analyse image and depth data from the Kinect. Spearman’s rho was used to assess intra-rater reliability and concurrent validity of the Kinect to evaluate foot posture, and a linear regression was used to examine the ability of the Kinect to predict total visual FPI score.

**Results:**

The Kinect demonstrated moderate to good intra-rater reliability for four FPI items of foot posture (ρ = 0.62 to 0.78) and moderate to good correlations with the 3DMA system for four items of foot posture (ρ = 0.51 to 0.85). In contrast, intra-rater reliability of visual FPI items was poor to moderate (ρ = 0.17 to 0.63), and correlations with the Kinect and 3DMA systems were poor (absolute ρ = 0.01 to 0.44). Kinect FPI items with moderate to good reliability predicted 61% of the variance in total visual FPI score.

**Conclusions:**

The majority of the foot posture items derived using the Kinect were more reliable than the traditional visual assessment of FPI, and were valid when compared to a 3DMA system. Individual foot posture items recorded using the Kinect were also shown to predict a moderate degree of variance in the total visual FPI score. Combined, these results support the future potential of the Kinect to accurately evaluate static foot posture in a clinical setting.

## Background

Abnormal foot posture and mechanics have long been associated with lower limb injuries [1-4]. For instance, clinical measures of a pronated foot posture, such as a low arch [5] and excessive navicular drop [6], have been retrospectively identified with knee injuries and anterior cruciate ligament injury respectively. Similarly, measures indicating a more supinated foot posture, such as a high arch [7] and rearfoot varus [3] have been retrospectively associated with stress fractures and patellofemoral pain respectively. Consequently, the measurement and classification of foot posture in a clinical setting has become a central focus of lower extremity medicine, and now is widely used to evaluate injury risk and monitor treatment efficacy.

Despite the existence of many different techniques to evaluate foot posture in the clinical setting, there is still disagreement as to which method is the most clinically useful [8]. Indeed, some studies have found contrasting results regarding the association between abnormal foot type and injury depending on the clinical technique employed [3,4,9,10], with some researchers arguing that these conflicting findings may be at least partly due to the lack of reliability and validity of many of these measures [11-13]. Moreover, the inability of many of the static measures of foot posture to predict dynamic function also calls in to question their clinical utility [14,15].

To address these concerns, Redmond, Crosbie and Ouvrier [16] developed a subjective measure of static foot posture termed the Foot Posture Index (FPI). The FPI is comprised of one palpatory and five visual criteria used to determine whether the foot is in a supinated, neutral or pronated position [16]. Research has reported that the FPI possesses acceptable intra-rater reliability [1,17,18], and the tool has been validated against both static and dynamic three-dimensional (3D) lower limb models [16]. However, despite these advantages, the subjective nature and limited five-point Likert-type scoring scale of the FPI may limit the tool’s precision, with some researchers suggesting that the results need to be interpreted with caution and may actually have limited value, especially in a research setting [18]. Consequently, there remains a need for an inexpensive, portable and accurate assessment tool that can quantifiably assess static foot posture, which could be implemented in a clinical setting for everyday patient assessment.

The Microsoft Kinect™ is an inexpensive and portable video game accessory that combines a video and infrared-sensing camera to create a 3D model of the body. Recent research has shown that the Kinect system is capable of creating this 3D human model with similar accuracy to expensive and complex 3D body scanning systems [19]. Similarly, early work has also shown promising results for the Kinect to evaluate gait velocity [20], hand and elbow movements [21] and anatomical landmark displacement and trunk angle [22] when compared to 3D motion analysis systems. Combined, these studies demonstrate that the Kinect is able to obtain some kinematic and anatomical mapping data with a similar degree of accuracy to more expensive 3D motion analysis and scanning systems [19,22]. Consequently, the Kinect may have the potential to objectively evaluate static foot posture in a clinical setting with more accuracy than the subjectively based FPI. This in turn may permit better injury prediction accuracy, increased measurement reliability and improved clinical utility. Therefore, this study aimed to evaluate whether the Kinect is able to reliably and validly evaluate static foot posture, as measured using the FPI. A secondary aim was to validate the Kinect-derived data with assessment of static foot posture using a 3D motion analysis system. Lastly, a third aim was to examine whether Kinect measures of foot posture were able to predict the variance in the total visual FPI score.

## Methods

### Participants

A convenience sample of 30 young, healthy males (age: 22.2 ± 3.2 years, height: 177.4 ± 5.0 cm, mass: 74.4 ± 4.7 kg, leg length - right limb: 92.9 ± 3.9 cm, knee width - right limb: 9.9 ± 0.7 cm, ankle width – right limb: 7.5 ± 0.3 cm) with no lower limb injuries in the prior two months volunteered to participate in this study. Only males were recruited due to the potential influence of menstrual cycle phase on lower limb function and tendon mechanical properties and the likely impact of this on test re-test reliability data [23,24]. Participants completed informed consent forms prior to testing and all procedures were approved (approval number 2012 47V) by the Australian Catholic University Human Research Ethics Committee.

### Procedure

This study used a concurrent validity, test re-test reliability design to determine if the Kinect can reliably and validly evaluate static foot posture, using items described for the FPI. This design validated the accuracy of measuring the FPI using the Kinect against the traditional visual observation of the FPI and a benchmark reference, the Vicon motion analysis system. Additionally, the repeated measures design was used to evaluate the reliability of measuring the FPI using the Kinect.

Eligible participants attended two testing sessions, seven days apart (median = 7; IQR = 5 to 9). Basic anthropometric data were recorded in the first session, such as height (cm), mass (kg), leg length (cm), knee width (cm) and ankle width (cm). Next, each participant’s foot posture was evaluated by visual observation (FPI), and by using both the Vicon and Kinect systems as described below. Participants were required to return approximately one week following the initial testing session where these foot posture measurements were repeated.

#### Assessment of the Foot Posture Index using visual observation

The Foot Posture Index (FPI) is a validated, subjective measure that is widely used in a clinical setting, and which is comprised of six measurements of the foot to evaluate static foot posture [16]. The FPI has been shown to possess good intra-rater reliability (ICC = 0.81 – 0.94), however inter-rater reliability has only been shown to be moderate to good (ICC = 0.53 – 0.79) [1,17,18]. The FPI has also been validated against an electromagnetic motion tracking system with results demonstrating that the six-item FPI total score predicted 64% of the variation (R^2^ = 0.64, p < 0.001) in static measurements [16].

The six-item version of the FPI includes evaluation of talar head palpation (FPI item 1), supra and infra lateral malleolar curvature (FPI item 2), calcaneal inversion/eversion (FPI item 3), talo-navicular joint bulging (FPI item 4), congruence of the medial longitudinal arch (FPI item 5), and forefoot abduction/adduction (FPI item 6) [16]. A five-point Likert-type scale is used to score each of these items, from −2 to +2, with zero being the central value or a neutral position and negative and positive values indicating a more supinated or pronated position respectively. A total FPI score of above +10 indicates a highly pronated foot posture, +6 to +9 indicates a pronated foot posture, 0 to +5 indicates a normal or neutral foot posture, -1 to −4 indicates a supinated foot posture while a score of −5 to −12 indicates a highly supinated foot posture [16].

Participants were told to assume a relaxed comfortable stance with their right foot on a straight line drawn on the ground. This line was orientated on the Y-axis of the laboratory to align the foot with the axes of the global coordinate system. Their foot was then positioned so that the posterior calcaneus and the interspace between the second and third metatarsophalangeal joints were bisecting the line, aligning the central axis of the foot with the axis of the laboratory reference frame. Scores for each of the six items of the FPI were recorded after visual observation of the participant’s right foot. This was completed on each of the two sessions, approximately one week apart, with the same rater recording visual FPI scores for every session who was blinded to any earlier scores. The rater in this study was a researcher who received three one-hour training sessions for the FPI prior to the study from an experienced podiatrist. The same researcher conducted all analyses of the Vicon and Kinect systems.

#### Assessment of the Foot Posture Index using the Vicon analysis system

An eight camera visible red Vicon 3D motion analysis system (Vicon, Oxford, United Kingdom) sampling at 200 Hz was used to determine each of the FPI items, with the exception of talar head palpation. To evaluate the static FPI using the Vicon system, a calibration wand was used to identify the items of the modified FPI. Following calibration, the system used the fixed markers on the wand to determine the position of the wand tip in 3D space [25]. Pointing the wand tip at specific anatomical landmarks, or tracing regions using the wand tip, may be used to provide a high degree of accuracy without the limitation of soft tissue artefact or marker placement inaccuracy [26]. Five different trials were captured, being lateral curvature, lateral and medial landmarks, rearfoot mesh and medial mesh trials as explained below.

To establish the lateral malleolar curvature (FPI item 2), the proximal and distal groove sizes were calculated from the virtual marker, and the proximal groove was subtracted from the distal groove. This was done by plotting the vertical and mediolateral position of the wand tip (in the anatomical reference frame) on a two-dimensional (2D) scatter plot for visualisation using the data from this trial. This created a graph that represented the lateral surface of the lower leg along a slice running from mid-shank to the plantar calcaneal fat pad. The distal groove was identified based on the minimum, or most medial, wand tip position below the malleolus, while the proximal groove was deemed the minimum value above the malleolus.

To assess calcaneal inversion/eversion (FPI item 3), a rearfoot mesh trial was used. The mesh was created by sweeping the wand tip across the rearfoot mediolaterally, gradually moving from the most inferior to superior point on the calcaneus, with the vertical distance between each sweep within 5 mm. The most posterior point of the calcaneus was found for each sweep, with this position identified by plotting the medial-lateral and anterior-posterior positions of the marker on a 2D scatter plot for visualisation. The single most posterior position of the wand tip in each medial-lateral sweep was then identified and extracted. The resultant angle of these points to the vertical was established using a least-square error linear fit to calculate calcaneal inversion/eversion.

Talo-navicular joint bulging (FPI item 4) was determined by using the medial landmarks trial, calculated by measuring the medial-lateral displacement of the navicular tuberosity in relation to the medial calcaneus. Congruence of the medial longitudinal arch (FPI item 5) was calculated from a medial mesh trial and split into two categories, arch height and arch peak. The wand tip was swept across the medial foot vertically, gradually moving from the medial calcaneus to the head of the first metatarsal. In this case, the most medial position of the wand tip during each vertical sweep was extracted, and was plotted on a 2D scatter plot for visualisation of the medial arch. These extracted points were then smoothed using a polynomial filter and the most superior point on the plot was deemed the arch height. Additionally, the anterior-posterior position of the arch height point was also expressed as a percentage distance from the calcaneus to the head of the first metatarsal, termed arch peak. Therefore, FPI item 5 was compared to two measures, arch height and arch peak, derived from the Vicon system.

Lastly, forefoot abduction/adduction (FPI item 6) used both the lateral and medial landmark trials. The medial-lateral displacement between the lateral calcaneus and the head of the fifth metatarsal was compared with the medial-lateral displacement between the medial calcaneus and the head of the first metatarsal. The medial displacement was then subtracted from the lateral displacement to give a score for forefoot abduction/adduction. Refer to Additional file 1 for further detail and images regarding the assessment and data analysis of foot posture using the Vicon 3D motion analysis system.

#### Assessment of the Foot Posture Index using the Microsoft Kinect™

Each of the FPI items, with the exception of talar head palpation, was also captured using a Microsoft Kinect camera. The video camera can record images at a variety of resolutions, with the resolution for this study set at 640×480 pixels. The infrared-sensing camera acts as a depth sensor that determines the distance of objects in front of the Kinect, and was used to obtain a calibrated depth map of this area at a resolution of 320×240 pixels. The precision of the depth map becomes exponentially worse as the distance from the Kinect increases, however it has been shown to possess a precision of < 3 mm at the range used in this study [27]. Additionally, although the individual pixel data is somewhat noisy, the use of a 2D median filter allows for accurate depth mapping to be performed [28].

To acquire the Kinect data, the device was firstly placed on the ground at a distance of 100 cm from where the participant was required to stand, and a calibration technique was performed to set the global reference frame [22]. In each position, the participant’s right foot was placed on a line drawn on the ground 100 cm from the Kinect. The items of the modified FPI (talar head palpation being excluded) were then acquired in lateral, posterior and medial views of the foot whilst the participant stood as still as possible. To allow the camera to see the medial aspect of their foot, the participant’s left foot was placed in a comfortable position behind them and they were instructed to keep their right shank perpendicular to the ground and to have approximately 50% of their body mass through their right foot.

Custom made LabVIEW software (National Instruments, U.S.A.) was used to collect and analyse the data from the Kinect using the Microsoft Software Development Kit (SDK) Beta 2 (Microsoft, U.S.A.). Data were sampled at the native frequency of the Kinect, which is irregular at ≈ 30Hz. The depth image was converted to the same resolution as the video image using interpolation, and the two images were aligned using a cross correlation function. The real world coordinates of the pixels in the video and depth images were determined using the calibration data extracted from the SDK. When anatomical landmarks or specific positions needed to be identified, these were located using the video image and the corresponding points on the depth map were extracted. For all values the median of five consecutive frames of Kinect data were used.

The lateral position trial was used to determine the lateral malleolar curvature (FPI item 2). Specifically, the video image was used to place cursors along a vertical line that had previously been drawn on the participant’s leg to represent the middle of the lateral aspect of their shank. The proximal and distal groove size was then calculated from the corresponding depth sensor data, and the proximal groove was subtracted from the distal groove. To remove noise and improve the accuracy of the depth data, a five point median filter with the centre positioned on the line drawn on the participant’s leg was applied to each vertical pixel row.

For calcaneal inversion/eversion (FPI item 3) the rearfoot position trial was used. The most posterior points of the calcaneus in each pixel row were determined from the depth sensor data and the resultant angle of these points was calculated against the vertical.

Talo-navicular joint bulging (FPI item 4) was determined from the medial trial by calculating the medial-lateral displacement of the navicular tuberosity in relation to the medial calcaneus. This was done through the identification of these landmarks by manually placing cursors on the video image and using the corresponding points on the depth map.

The medial trial was also used to determine the congruence of the medial longitudinal arch (FPI item 5) in a similar way to the Vicon analysis. The vertical position of the point in each vertical column of the depth map pixels was identified and plotted on a 2D scatter plot along its anterior-posterior position. The maximum vertical position was deemed arch height, and the anterior-posterior position of this was termed the arch peak. The arch peak was calculated relative to the distance between the calcaneus and head of the first metatarsal, and expressed as a percentage from the calcaneus. Therefore, similar to the Vicon analysis, FPI item 5 was compared to two measures derived from the Kinect, arch height and arch peak.

Lastly, forefoot abduction/adduction (FPI item 6) used both the lateral and medial position trials. The medial-lateral displacement between the lateral calcaneus and the head of the fifth metatarsal was compared with the medial-lateral displacement between the medial calcaneus and the head of the first metatarsal. These points were first identified manually on the video image and the corresponding points on the depth image were extracted for analysis. The medial displacement was then subtracted from the lateral displacement. Refer to Additional file 2 for further detail and images regarding the assessment and data analysis of foot posture using the Kinect system.

### Statistical analysis

All data were imported to and analysed using the Statistical Package for Social Sciences (SPSS) version 19.0. Data were initially assessed for normality using the Shapiro-Wilks test (*p* > 0.05), however the majority of data were not normally distributed hence non-parametric statistics were used.

To examine test-retest reliability of the FPI and Kinect data, Spearman’s rho was calculated for each FPI item (visual observation, Vicon and Kinect analysis of FPI) and a rank transformed intraclass correlation coefficient (ICC) (2,1) was used for total FPI score (visual observation of FPI only), which is consistent with previous research [29]. To determine the concurrent validity of the Kinect to measure foot posture, Spearman’s rho, the non-parametric measure of association between two variables, was used [30]. Data obtained from the Kinect were compared to scores obtained from both the visual observation of the FPI as well as those derived from the Vicon system for each of the FPI items recorded. Additionally, the visual observation of the FPI was also compared to FPI data obtained from the Vicon system. Based on the thresholds provided by Portney and Watkins [31], poor correlations were interpreted as a fair or below-fair relationship (< 0.50) and acceptable correlations were deemed reliable and valid as a moderate to good (0.50 – 0.75) or good to excellent (> 0.75) relationship for all reliability and validity analyses.

To further examine the agreement between the Vicon and Kinect systems for each FPI item, 95% limits of agreement (LOA) and Bland-Altman plots were also used if the mean difference between systems was normally distributed [32]. The LOA method uses the mean difference between the measures and the standard deviation of the differences, and states that the plots should fall between ±1.96SD of the mean difference [32]. The Bland-Altman method plots the difference between the FPI items recorded by the Vicon and Kinect systems against the mean of the two measures. Visual inspection for any fixed or proportional bias were performed on the Bland-Altman plots. Finally, to determine if the Vicon and Kinect systems were able to predict the total scores obtained on the clinical FPI observation, a linear regression was used on rank transformed data. The reliable items from the Vicon and Kinect systems obtained in the first testing session were entered as the independent variables with the total FPI score as the dependent variable. The total FPI score can be compared against the continuous Vicon and Kinect data as the summation of the FPI items to obtain a total FPI score results in continuous data [29]. If a measure demonstrated significance (*p* < 0.05) in this regression, a Spearman’s rho correlation was used to determine the strength of the association between the measure and total FPI score.

## Results

### Reliability

Descriptive statistics and intra-rater reliability for each of the FPI items measured by the visual FPI, Kinect and Vicon systems are presented in Table 1. Reliability of the visual FPI items was mixed, with the ICC value for the total visual FPI score demonstrating good to excellent intra-rater reliability (ICC = 0.87), however moderate to good reliability was shown for lateral curvature and congruence of the medial longitudinal arch (ρ = 0.52 to 0.63), and poor reliability for talar head palpation, calcaneal inversion/eversion, talo-navicular joint bulging and forefoot abduction/adduction (ρ = 0.17 to 0.42). The Spearman’s rho for the Kinect indicates good to excellent intra-rater reliability for lateral curvature (ρ = 0.78), with moderate to good reliability for talo-navicular joint bulging, arch height and arch peak (ρ = 0.62 to 0.72) and poor reliability for forefoot abduction/adduction and calcaneal inversion/eversion (ρ = 0.21 to 0.30). The intra-rater reliability for Vicon items were similar, with good to excellent reliability for talo-navicular joint bulging and arch height (ρ = 0.78 to 0.79), with moderate to good reliability for lateral curvature and forefoot abduction/adduction (ρ = 0.55 to 0.73) and poor reliability for arch peak and calcaneal inversion/eversion (ρ = 0.13 to 0.37).

**Table 1:** Intra-rater reliability of the visual FPI, Kinect and Vicon systems for each item of the FPI

	**FPI**	**Kinect**	**Vicon**
FPI 1 Talar head			
Day 1 median (IQR)	0 (0 to 1)		
Day 2 median (IQR)	1 (0 to 1)	N/A	N/A
Spearman’s rho	0.38*		
FPI 2 Lateral curvature			
Day 1 median (IQR)	0 (0 to 1)	−1.55 (−3.40 to 0.75)	−1.32 (−5.06 to 0.93)
Day 2 median (IQR)	0 (0 to 1)	−0.97 (−4.29 to 0.74)	−2.83 (−4.06 to 0.51)
Spearman’s rho	0.52*	0.78*	0.73*
FPI 3 Calcaneal inv/ev			
Day 1 median (IQR)	0 (0 to 1)	10.03 (7.54 to 11.71)	9.77 (6.82 to 12.98)
Day 2 median (IQR)	1 (0 to 1)	11.34 (9.32 to 14.26)	10.73 (7.57 to 12.18)
Spearman’s rho	0.42*	0.30	0.37
FPI 4 TNJ bulging			
Day 1 median (IQR)	1 (0 to 1)	−17.00 (−22.00 to −13.00)	−12.93 (−15.86 to −8.52)
Day 2 median (IQR)	1 (0 to 1)	−16.50 (−19.25 to −13.00)	−11.63 (−16.22 to −8.71)
Spearman’s rho	0.17	0.62*	0.79*
FPI 5 Congruence of MLA/Arch height			
Day 1 median (IQR)	1 (0 to 2)	49.35 (39.94 to 61.36)	26.75 (22.19 to 31.12)
Day 2 median (IQR)	1 (0 to 1)	46.28 (40.33 to 58.88)	28.82 (23.86 to 32.72)
Spearman’s rho	0.63*	0.72*	0.78*
FPI 5 Arch peak			
Day 1 median (IQR)		17.65 (15.69 to 32.65)	42.67 (38.25 to 45.03)
Day 2 median (IQR)	N/A	24.20 (15.56 to 35.76)	42.41 (34.59 to 46.53)
Spearman’s rho		0.66*	0.13
FPI 6 Forefoot ab/add			
Day 1 median (IQR)	1 (0 to 2)	−3.00 (−6.00 to 3.00)	2.87 (−0.71 to 7.88)
Day 2 median (IQR)	1 (0.75 to 1)	0.00 (−6.00 to 6.50)	4.19 (−1.15 to 8.75)
Spearman’s rho	0.29	0.21	0.55*
FPI total score			
Day 1 median (IQR)	3 (2 to 6)		
Day 2 median (IQR)	4 (2 to 5.25)	N/A	N/A
ICC	0.87*		

Note: * *p* < 0.05; *FPI* = Foot Posture Index; *IQR* = interquartile range; *TNJ* = talo-navicular joint; *MLA* = medial longitudinal arch; *ICC* = Intraclass correlation coefficient.

### Validity

Concurrent validity between the visual FPI, Kinect and Vicon systems are displayed in Table 2. Results of the validity analysis indicate a good to excellent correlation between the Kinect and Vicon items of lateral curvature (ρ = 0.85). Moderate to good correlations were shown for talo-navicular joint bulging, arch height and forefoot abduction/adduction (ρ = 0.51 to 0.74) and poor correlations were found for arch peak and calcaneal inversion/eversion (ρ = 0.30 to 0.34). The concurrent validity results demonstrated that all individual visual FPI items correlated poorly with the corresponding items recorded using either the Kinect or Vicon systems, with all of the items indicating little to fair relationships (absolute ρ = 0.01 to 0.44).

**Table 2:** Concurrent validity between systems for each FPI item

		**Kinect-FPI ρ**	**Kinect-Vicon ρ**	**Vicon-FPI ρ**
**FPI item**	**Instrumented item**			
FPI 2 Lateral curve	Lateral curve	−0.03	0.85*	−0.01
FPI 3 Calcaneal inv/eversion	Calcaneal inv/eversion	0.36*	0.34	0.44*
FPI 4 TNJ bulging	TNJ bulging	−0.34	0.74*	−0.35
FPI 5 Congruence of MLA	Arch height	−0.01	0.51*	−0.20
	Arch peak	−0.36	0.30	−0.44*
FPI 6 Forefoot ab/adduction	Forefoot ab/adduction	−0.14	0.57*	−0.19

Note: * *p* < 0.05; *FPI* = Foot Posture Index; ρ = Spearman’s rho; *TNJ* = talo-navicular joint; *MLA* = medial longitudinal arch.

**Figure 1:**
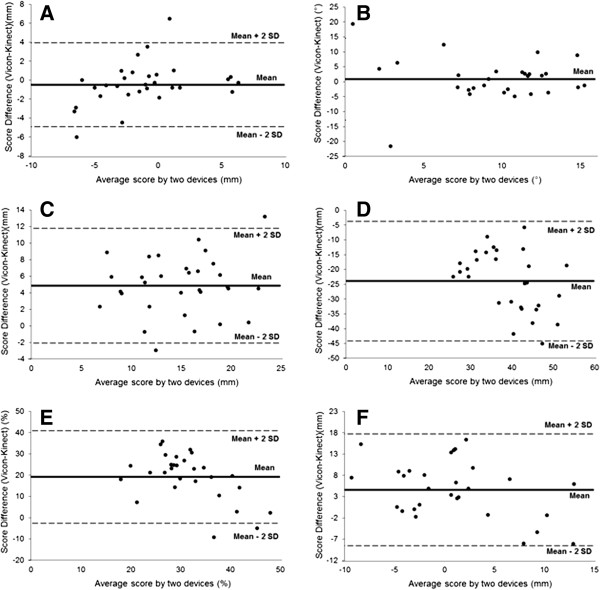
**Bland-Altman plots demonstrating the agreement between the Kinect and Vicon systems for each FPI item. A**: Lateral curvature (FPI item 2). **B**: Calcaneal inversion/eversion (FPI item 3). **C**: Talo-navicular joint bulging (FPI item 4). **D**: Arch height (FPI item 5). **E**: Arch peak (FPI item 5). **F**: Forefoot abduction/adduction (FPI item 6). Note: LOA were not calculated for B due to the mean difference not being normally distributed.

Bland-Altman plots were also used to indicate the agreement between the Kinect and Vicon systems for each FPI item (Figure 1). The LOA method was calculated for all items except for calcaneal inversion/eversion (FPI item 3) due to the mean difference between the systems not being normally distributed for this item. Fixed biases were evident between devices for the arch height (Figure 1D) and arch peak items (Figure 1E), whereas no obvious relationship between the difference and the mean was observed for any item. These plots demonstrate poor absolute agreement between the two devices.

The Kinect items with moderate to excellent reliability (ρ > 0.50) that were entered into the regression model were lateral curvature, talo-navicular joint bulging, arch height and arch peak. The regression analysis showed that the reliable Kinect items explained 61% (r = 0.78, R^2^ = 0.61, F = 10.08, *p* < 0.001) of the variance in the total visual FPI score. However, the only item that independently demonstrated significance (*p* < 0.05) was talo-navicular joint bulging. Similarly, the Vicon items with moderate to excellent reliability (ρ > 0.50) (lateral curvature, talo-navicular joint bulging, arch height and forefoot abduction/adduction) explained 58% (r = 0.76, R^2^ = 0.58, F = 8.2, *p* < 0.001) of the variance in the total visual FPI score, with only talo-navicular joint bulging again independently demonstrating significance (*p* < 0.05). Correlations between talo-navicular joint bulging, recorded using the Kinect and Vicon systems, and the total visual FPI score were shown to be good to excellent (ρ = 0.75, *p* < 0.001; ρ = 0.74, *p* < 0.001 respectively).

## Discussion

This study was the first to examine the reliability and validity of the Microsoft Kinect to evaluate static foot posture. The Kinect demonstrated moderate to good reliability for four out of six items of the modified FPI (lateral malleolar curvature, talo-navicular joint bulging and medial arch height and peak). Additionally, the Kinect also displayed moderate to good concurrent validity for four items of the FPI when compared to the Vicon 3D analysis system (lateral malleolar curvature, talo-navicular joint bulging, medial arch height and forefoot abduction/adduction). However the relationship between Kinect and visual FPI items was found to be poor. Regression analysis revealed that the Kinect FPI items with moderate to good reliability were able to predict 61% of the variance in the total visual FPI score, with the only significant variable (talo-navicular joint bulging) also demonstrating a good to excellent relationship with the total visual FPI score. The advantage of the Kinect in comparison with the individual items of the visual FPI is that it provides quantified data on a continuous scale rather than on a limited ordinal scale. Although somewhat mixed, these results support the future potential use of the Kinect as a tool to assess static foot posture in a clinical setting.

The Kinect showed improved intra-rater reliability for individual items of the FPI when compared with the visual FPI observations. Specifically, individual FPI items recorded by the Kinect showed moderate to good reliability whereas visual FPI items demonstrated poor to moderate intra-rater reliability, which is consistent with previous research [29]. The total visual FPI score demonstrated good to excellent intra-rater reliability, which has also been reported previously [1,17,18]. The improved item reliability found with the Kinect could be attributed to the continuous data of the Kinect which, compared with the limited ordinal scale of the FPI, potentially provides improved accuracy in the evaluation of foot posture.

Similar to the finding of improved reliability of FPI items recorded using the Kinect, Vicon FPI items also demonstrated superior reliability when compared to the visual assessment of FPI. However, there was some variation in reliability levels between the systems. For instance, arch peak, which is part of the congruence of the medial longitudinal arch item of the FPI, demonstrated moderate to good intra-rater reliability for the Kinect system whilst poor reliability was shown for the Vicon system. In contrast, forefoot abduction/adduction demonstrated moderate to good reliability for the Vicon system whereas poor reliability was shown for the Kinect system. Furthermore, the item calcaneal inversion/eversion demonstrated poor intra-rater reliability for both systems.

The variable reliability results for the Kinect and Vicon systems may be partly due to the different data collection methods employed, with the Kinect system using depth data and the Vicon system using a wand to locate landmarks and trace over regions of the foot in 3D space. For instance, the precision along the longitudinal axis of the foot of the medial mesh techniques used in the Vicon analysis of the FPI, which involved performing sweeping movements over the medial surface of the foot using the wand tip, was poor relative to the depth data from the Kinect for measurement of the arch peak along the longitudinal axis of the foot. The distance between each sweep may have been too large (approximately 10 mm at the top of the arch), whilst the Kinect was able to measure the 3D position of the arch in each pixel with a longitudinal axis precision of approximately 3 mm. Furthermore, the wand tip may depress the soft tissue of the foot as the sweeps were performed along the medial arch. This may limit the ability to identify the position of the arch peak due to the varying compressions in the medial-lateral plane of the soft tissue, which would not affect the measure of arch height in the vertical plane. Instrumenting the wand and controlling force application during the assessment may have reduced this error, however during our pilot testing this proved difficult to control via feedback given that the forces applied through the wand tip were quite low (< 5 Newtons).

Additionally, the Kinect demonstrated poor reliability for the FPI item of forefoot abduction/adduction, which may be attributed to errors in visual anatomical landmark identification. The reliability for this item may be improved by identifying the furthest point of the rearfoot to the Kinect compared to the closest point of the forefoot to the Kinect. Finally, calcaneal inversion/eversion also demonstrated poor reliability for both the Kinect and Vicon systems. As discussed for the Vicon assessment of arch peak, the precision of the mesh technique for evaluation of calcaneal inversion/eversion may have been affected by the distance between each sweep and the varying compression of the soft tissue. Similarly, the poor reliability found for the Kinect assessment of calcaneal inversion/eversion may have been due to errors in visual estimation of calcaneal position, as suggested previously for forefoot abduction/adduction. Indeed, difficulties in visually estimating rearfoot position have been highlighted previously [33], and given the present study visually assessed a depth image rather than the actual foot, it is likely that this technique may have led to further errors in the evaluation of this item.

Validity analysis revealed that all individual items of the FPI derived from visual observations were poorly correlated with items from both the Kinect and 3D motion analysis system. This inability to correlate the visual FPI with other measures of foot posture is consistent with Scharfbillig et al. [34], who reported that four items of the FPI were poorly correlated with radiographic measures, which they partly attributed to a lack of agreement between bony architecture and the overlying skin. Given each item of the visual FPI has only five possible scores, this limited spread of data will reduce the likelihood of finding strong relationships when compared with a continuous set of data such as depth or radiological-based measures. Further, the reduced reliability of the individual visual FPI items when compared to both the Kinect and Vicon FPI items may limit the appropriateness of the visual inspection of foot posture as a concurrent validity measure. In contrast, mostly moderate to good correlations were found between the Kinect and Vicon, which is likely due to the two analysis systems using continuous data and having the same outcome measures. Bland-Altman plots revealed poor absolute agreement between the devices, although this may be explained by the different scales used by the Kinect and Vicon.

The Kinect items with moderate to excellent reliability were shown to predict 61% of the variance in the total visual FPI score. Similarly, the Vicon items with moderate to excellent reliability predicted 58% of the variance in the total FPI score derived from visual observations. In both cases, talo-navicular joint bulging was the only item entered into the regression model to independently demonstrate significance (*p* < 0.05). Although no previous study has investigated the ability of objective 3D measures of foot posture or an individual analysis of each of the FPI items to predict total FPI score, Redmond et al. [16] reported that total FPI score was able to predict 64% of the variance in a measure of ankle joint position in 3D space. The validation of the Kinect in explaining a high proportion of the variance in total visual FPI scores suggests the potential feasibility of the Kinect and custom analysis software to be further refined to classify overall foot posture. Indeed, given the greater reliability of the individual items of the FPI derived from the Kinect, and the greater similarity in Kinect FPI items to FPI items from the 3D analysis system, future studies may attempt to develop a total Kinect FPI-type score that could be used to classify foot posture with greater accuracy and reliability compared to the FPI derived from visual observations.

Interestingly, one item of the FPI, talo-navicular joint bulging, demonstrated good to excellent correlations with the total visual FPI score when measured by the Kinect. This may indicate the potential of this particular item of the FPI derived from the Kinect to be used as a stand-alone measure to classify foot posture. Similarly, other research has reported that clinical measures of the midfoot strongly correlated with radiographic measures of foot posture [35]. Future research should further examine the agreement between the measure of talo-navicular joint bulging derived from the Kinect and total FPI score.

A limitation of the current study is the lack of a total score for the Kinect FPI items. Given the different scales used within each FPI item for the Kinect, this made the generation of a total FPI-type score and foot posture classification problematic. Future research may wish to first implement techniques, such as multiple regressions, to derive a total FPI-type score and foot classification from the Kinect items and secondly to collect a comprehensive data set of the full range of foot posture using the Kinect, from highly supinated to highly pronated. Additionally, future research should examine the inter-rater reliability of the Kinect to evaluate foot posture given previous research has shown poor inter-rater reliability of the FPI based on visual observation [1,18]. If inter-rater reliability was found to be superior, this may further the potential use of the Kinect as a tool to assess static foot posture in a clinical setting. Although mostly moderate to excellent correlations were found between the Kinect and the 3D motion analysis system, another potential limitation may have been the use of the Vicon system as a benchmark reference. This is supported by the poor test re-test reliability of the Vicon data in evaluating two FPI items, and may be partially explained by the limited accuracy from the distance between sweeps along the longitudinal axis of the foot and from soft tissue deformation as explained previously. In contrast, previous research has shown that a 3D foot scanner is able to reliably and accurately provide a 3D digital representation of the foot [36,37]. Although such a tool may provide a more appropriate benchmark reference with which to compare the Kinect, the Vicon system was used in this study due to the need of further research to develop standardised 3D foot scanning protocols for evaluating foot posture and morphology, and the limited availability of such systems. Future studies may wish to examine the concurrent validity of the Kinect to evaluate static foot posture when compared to 3D foot scanners. Furthermore, the generalizability of the study may be comprised due to the male only participants and the use of a novice rater for assessment. To attain generalised results, future research is required using many raters with larger participant numbers.

## Conclusions

This study found that four foot posture items derived using the Microsoft Kinect demonstrated good intra-rater reliability and four items were valid when compared to a 3D analysis system. In contrast, poor reliability and validity was shown for the visual inspection of individual FPI items. Items of foot posture recorded using the Kinect were also shown to predict a moderate degree of variance in the total FPI score derived from visual observations. Future research should consider developing a total FPI-type score and foot posture classification using the Kinect FPI items. Combined, these results support the future potential of the Kinect to accurately evaluate static foot posture in a clinical setting.

## Competing interests

This study was funded in part by ASICS Oceania. All authors have received funding from ASICS Oceania either directly or indirectly via research grants or employment. Author RC designed the software and may at some stage release it either for free or at a cost.

## Authors’ contributions

Authors BM, KP and RC were involved in all aspects of the study. Author AM was involved in the pilot testing, data collection and drafting stage of the study. Authors SB and AB were involved in the preliminary design and drafting stages of the study. All authors read and approved the final manuscript.

## Supplementary Material

Additional file 1 **Assessment of the Foot Posture Index using the Vicon analysis system.** Procedure and data analysis for the assessment of foot posture using the Vicon motion analysis system. Click here for file

Additional file 2 **Assessment of the Foot Posture Index using the Microsoft Kinect™.** Procedure and data analysis for the assessment of foot posture using the Kinect. Click here for file
