# Supplementary material for: Reliability and validity of the Microsoft Kinect for evaluating static foot posture
Source: J Foot Ankle Res. 2013 Apr 8;6:14. doi: 10.1186/1757-1146-6-14 (PMC3639226; doi:10.1186/1757-1146-6-14)
Supplement: Additional file 1 — Assessment of the Foot Posture Index using the Vicon analysis system. Procedure and data analysis for the assessment of foot posture using the Vicon motion analysis system. [file 1757-1146-6-14-S1.docx]

**Reliability and validity of the Microsoft Kinect for evaluating static foot posture**

Benjamin F. Mentiplay^1^, Ross A. Clark^1^, Alexandra Mullins^1^, Adam L. Bryant^2^, Simon Bartold^2^, Kade Paterson^1§^

**Additional File 1**

Title: Assessment of the Foot Posture Index using the Vicon analysis system

Description: Procedure and data analysis for the assessment of foot posture using the Vicon motion analysis system.

**Procedure**

An eight camera visible red Vicon three-dimensional (3D) motion analysis system (Vicon, Oxford, United Kingdom) sampling at 200 Hz was used to determine each of the FPI items, with the exception of talar head palpation. Marker-based analysis systems are considered the gold standard of laboratory 3D motion analysis with numerous studies employing it to assess the concurrent validity of potentially clinically feasible devices [1-3].

The most common method of evaluating lower extremity mechanics using 3D systems involves placing small (~14mm) spherical retro-reflective markers on the skin overlying important anatomical landmarks (e.g. lateral malleolus). However, previous research has reported that soft tissue artefact and anatomical landmark misplacement are well-known sources of error when using this technique [4-7]. Therefore, given the dependent variable under investigation was static foot posture, and as such anatomical markers were not required to track segments during dynamic motion, this study used a calibration wand to identify the location of lower extremity anatomical landmarks. This wand has five small (14mm) retro-reflective markers attached along the two axes, and a pointed tip was added at the end of one axis. Following calibration, the system uses the fixed markers on the wand to determine the exact position of the wand tip in 3D space [8]. As such, pointing the wand tip at specific anatomical landmarks, or tracing regions using the wand tip, may be used to provide a high degree of accuracy without the limitation of soft tissue artefact or marker placement inaccuracy. This calibration method using the wand has been reported to have very high accuracy with error of less than 1mm [9].

To evaluate the static FPI using the Vicon system, the calibration wand (see Figure 1) was used to identify the items of the modified FPI (talar head palpation was excluded). For each trial, the participant’s right foot was placed on a line drawn on the ground as described above. To measure the lateral malleolar curvature of each participant, the wand tip was first used to trace inferiorly down the lateral shank, starting at approximately half way between the knee and ankle joints and finishing at the ground. To improve the accuracy of the trace, a flexible plastic ruler was adhered to the skin along this line, with the wand tip traced along the border in the vertical direction. The most medial and lateral points both above and below the lateral malleolus were used to determine the superior and inferior curvature of the lateral malleolus (FPI item 2) as outlined below.

**
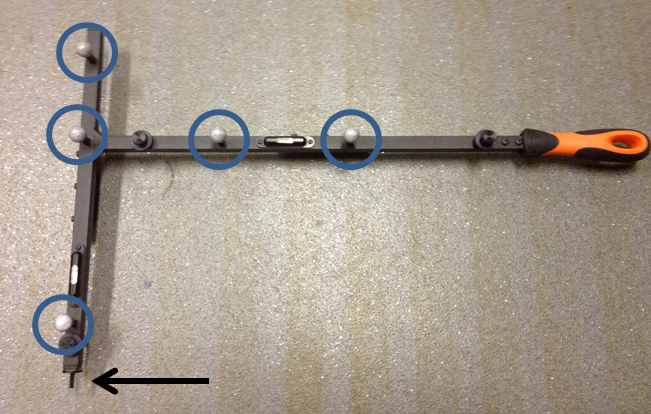
Figure 1. Calibration wand**. Circles: Five retro-reflective markers. Arrow: Wand tip used to trace over the foot.

Next, the second and third trials were used to identify lateral and medial foot landmarks. For the lateral landmarks (trial two), the wand was successively placed on the posterior calcaneus, lateral malleolus, lateral calcaneus, styloid process of the fifth metatarsal, head of the fifth metatarsal and the interspace between the second and third metatarsophalangeal joints. To identify the medial landmarks (trial three), the wand was placed on the posterior calcaneus, medial malleolus, medial calcaneus, navicular tuberosity, head of the first metatarsal and the interspace between the second and third metatarsophalangeal joints. For the calcaneus landmarks, the posterior calcaneus was identified as the most posterior point 30mm off the ground, and the medial and lateral calcaneus were identified as 30mm off the ground and 30mm anterior from the most posterior aspect of the calcaneus on the medial and lateral aspects respectively. The wand was positioned on each of these landmarks for approximately five seconds and then withdrawn approximately one metre before identifying the subsequent landmark. These combined landmarks (i.e. the medial and lateral landmarks in trials two and three) were used to establish forefoot abduction/adduction (FPI item 6), whilst the medial landmarks were also used to determine the talo-navicular joint bulging (FPI item 4), as outlined below. All landmarks were identified before data collection using a permanent marker.

For the fourth trial, the wand was traced over the rearfoot of the participant to determine frontal plane calcaneal position (i.e. varus/valgus). The wand was initially placed on the most inferior lateral aspect of the calcaneus then moved around the posterior aspect to the most inferior medial aspect. The wand was then moved slightly superiorly and the process repeated in the reverse direction (i.e. medial to lateral around the posterior aspect of the calcaneus), then moved superiorly and repeated again until eventually finishing on the most superior aspect of the calcaneus. This rearfoot ‘mesh’ was used to determine the calcaneal inversion/eversion angle (FPI item 3) as described below.

For the fifth trial, the wand was traced over the medial aspect of the foot to define the congruence of the medial longitudinal arch (FPI item 5). The wand was initially placed on the most inferior and posterior point of the medial aspect of the foot below the medial calcaneus landmark defined previously, then moved superiorly up the foot to the most superior aspect of the medial arch. The wand was then moved inferiorly down to the ground and this process repeated, moving the wand from the ground up to the most superior aspect of the arch, whilst moving more anteriorly each time until the wand was on the head of the first metatarsal. The first, fourth and fifth trials were captured twice to allow for unexpected errors. This process was completed on each of the two sessions, approximately one week apart. Figure 2 shows each position for Vicon assessment of the FPI.

**
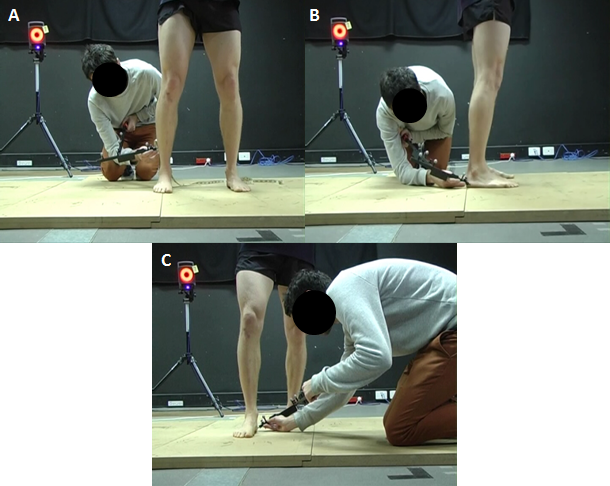
Figure 2. Three positions of the right foot for Vicon assessment of foot posture.** A: Lateral position of the right foot used for the lateral malleolar curvature and lateral landmarks trials. B: Posterior position of the right foot used for the rearfoot mesh trial. C: Medial position of the right foot used for the medial mesh and medial landmarks trials.

**Data Analysis**

Foot posture data obtained from the Vicon analysis system was recorded using Vicon Nexus software (version 1.7.1). Each Vicon trial was labelled in Vicon BodyBuilder software (version 3.6.1) using a custom marker set that included the five wand markers and any gaps in the data were filled based on the known position of the marker relative to the wand segment using Vicon Nexus software. A custom model was then run for each trial to create a virtual marker that represented the movement of the wand tip.

To establish the lateral malleolar curvature (FPI item 2), the proximal and distal groove sizes were calculated from the virtual marker, and the proximal groove was subtracted from the distal groove with a negative number indicating a more pronated foot posture. This was done by plotting the vertical and mediolateral position of the wand tip (in the anatomical reference frame) on a 2D scatter plot for visualisation using the data from this trial. This created a graph that represented the lateral surface of the lower leg along a slice running from mid-shank to the fat pad. The distal groove was identified based on the minimum, or most medial, wand tip position below the malleolus, while the proximal groove was deemed the minimum value above the malleolus. Consistent with the visual FPI scoring, a deeper proximal compared to distal groove was interpreted as a more pronated position. An example of a scatter plot with identified landmarks is provided in Figure 3.

**
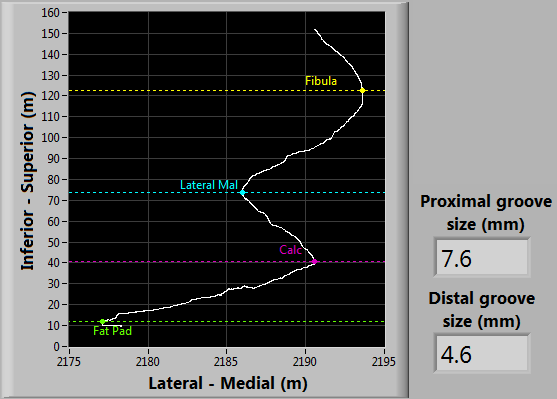
Figure 3. Vicon analysis: a trace of the lateral malleolar curvature (FPI item 2).**

To assess the calcaneal inversion/eversion (FPI item 3), the rearfoot mesh trial was used. The most posterior point of the calcaneus was found for each medial-lateral sweep of the wand tip with this position identified by plotting the medial-lateral and anterior-posterior positions of the marker on a 2D scatter plot for visualisation. The single most posterior position of the wand tip in each medial-lateral sweep was then identified and extracted. The resultant angle of these points to the vertical was established using a least-square error linear fit to calculate calcaneal inversion/eversion. A large positive angle indicates a pronated position whereas a large negative angle is indicative of a supinated position. An example of this is provided in Figure 4.


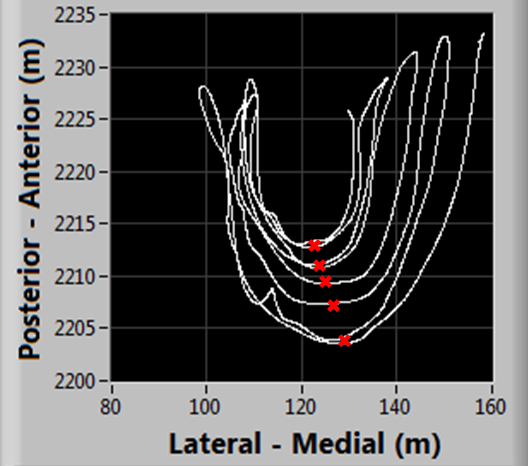


**Figure 4. Vicon analysis: a plot representing the sweeps around the rearfoot in a proximal view that were used to assess calcaneal inversion/eversion (FPI item 3).** The crosses demonstrate the most posterior point of each sweep.

Talo-navicular joint bulging (FPI item 4) was determined by using the medial landmarks trial, calculated by measuring the medial-lateral displacement of the navicular tuberosity in relation to the medial calcaneus. The position of the first metatarsal head was not used as a reference point due to the potential medial deviation of this joint in conditions such as hallux valgus. A more medial position of the navicular tuberosity compared to the medial calcaneus represented a more pronated position.

Congruence of the medial longitudinal arch (FPI item 5) was calculated from the medial mesh trial (and split in to two categories, arch height and arch peak) by finding the most medial aspect of the arch as the wand moved anteriorly. This technique was similar to that of the calcaneal inversion/eversion assessment, in that a single position during each full sweep was extracted for analysis. In this case, the most medial position of the wand tip during each vertical sweep was used, and was plotted on a 2D scatter plot with the location of this peak in the anterior-posterior axis used for visualisation of the medial arch. These extracted points were then smoothed using a polynomial filter and the most superior point on the plot was deemed the arch height. Additionally, the position of the point of the arch in the anterior-posterior axis was determined and expressed as a percentage distance from the calcaneus to the head of the first metatarsal. Therefore, FPI item 5 was compared to two measures, arch height and arch peak, derived from the Vicon system, which gave a total of six outcome measures from the Vicon measure of static foot posture. A more pronated position was one with a lower arch height and an anteriorly located peak indicated by a higher percentage. An example of this item is provided in Figure 5.

**
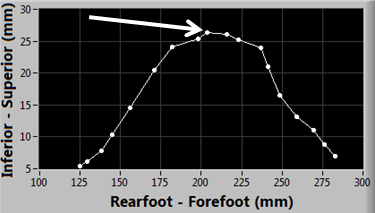
Figure 5. Vicon analysis: representation of the congruence of the medial longitudinal arch (FPI item 5).** Arrow: The position of the arch peak used for both measures of the congruence of the medial longitudinal arch (FPI item 5).

Lastly, forefoot abduction/adduction (FPI item 6) used both the lateral and medial landmarks trials. The medial-lateral displacement between the lateral calcaneus and the head of the fifth metatarsal was compared with the medial-lateral displacement between the medial calcaneus and the head of the first metatarsal. The medial displacement was subtracted from the lateral displacement, thus giving a higher score for forefoot abduction and indicating a more pronated foot posture.

**References**

1. Pomeroy VM, Evans E, Richards JD: **Agreement between an electrogoniometer and motion analysis system measuring angular velocity of the knee during walking after stroke.** *Physiotherapy* 2006, **92:**159-165.

2. Clark RA, Pua Y-H, Fortin K, Ritchie C, Webster KE, Denehy L, Bryant AL: **Validity of the Microsoft Kinect for assessment of postural control.** *Gait Posture* 2012, **36:**372-377.

3. Chung PYM, Ng GYF: **Comparison between an accelerometer and a three-dimensional motion analysis system for the detection of movement.** *Physiotherapy* 2012, **98:**256-259.

4. Leardini A, Chiari L, Della Croce U, Cappozzo A: **Human movement analysis using stereophotogrammetry: Part 3: Soft tissue artifact assessment and compensation.** *Gait Posture* 2005, **21:**212-225.

5. Lucchetti L, Cappozzo A, Cappello A, Croce UD: **Skin movement artefact assessment and compensation in the estimation of knee-joint kinematics.** *J Biomech* 1998, **31:**977-984.

6. Holden JP, Orsini JA, Siegel KL, Kepple TM, Gerber LH, Stanhope SJ: **Surface movement errors in shank kinematics and knee kinetics during gait.** *Gait Posture* 1997, **5:**217-227.

7. Della Croce U, Leardini A, Chiari L, Cappozzo A: **Human movement analysis using stereophotogrammetry: Part 4: Assessment of anatomical landmark misplacement and its effects on joint kinematics.** *Gait Posture* 2005, **21:**226-237.

8. Cappozzo A, Catani F, Della Croce U, Leardini A: **Position and orientation in space of bones during movement: anatomical frame definition and determination.** *Clin Biomech* 1995, **10:**171-178.

9. Donati M, Camomilla V, Vannozzi G, Cappozzo A: **Anatomical frame identification and reconstruction for repeatable lower limb joint kinematics estimates.** *J Biomech* 2008, **41:**2219-2226.
