# Supplementary material for: Reliability and validity of the Microsoft Kinect for evaluating static foot posture
Source: J Foot Ankle Res. 2013 Apr 8;6:14. doi: 10.1186/1757-1146-6-14 (PMC3639226; doi:10.1186/1757-1146-6-14)
Supplement: Additional file 2 — Assessment of the Foot Posture Index using the Microsoft Kinect™. Procedure and data analysis for the assessment of foot posture using the Kinect. [file 1757-1146-6-14-S2.docx]

**Reliability and validity of the Microsoft Kinect for evaluating static foot posture**

Benjamin F. Mentiplay^1^, Ross A. Clark^1^, Alexandra Mullins^1^, Adam L. Bryant^2^, Simon Bartold^2^, Kade Paterson^1§^

**Additional File 2**

Title: Assessment of the Foot Posture Index using the Microsoft Kinect^TM^

Description: Procedure and data analysis for the assessment of foot posture using the Kinect.

**Procedure**

Each of the FPI items, with the exception of talar head palpation, was also captured using a Microsoft Kinect camera. The Kinect is an inexpensive and portable video game accessory that combines a video and infrared-sensing camera to create a three-dimensional model of the body (see Figure 1). The video camera can record images at a variety of resolutions, with the resolution for this study set at 640x480 pixels. The infrared-sensing camera acts as a depth sensor that determines the distance of objects in front of the Kinect, and was used to obtain a calibrated depth map of this area at a resolution of 320x240 pixels. The precision of the depth map becomes exponentially worse as the distance from the Kinect increases, however it has been shown to possess a precision of < 3mm at the range used in this study [1]. Additionally, although the individual pixel data is somewhat noisy, the use of a 2D median filter allows for accurate depth mapping to be performed [2].

**
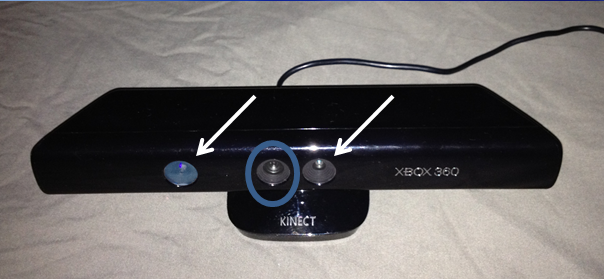
Figure 1. Microsoft Kinect.** Circle: Standard video camera. Left Arrow: Infrared emitter. Right Arrow: Infrared sensing camera.

To acquire the Kinect data, the device was firstly placed on the ground at a distance of 100cm from where the participant was required to stand, and a calibration technique was performed to set the global reference frame [3]. A background extraction trial was then recorded prior to the participant entering the data collection area. In each position, the participant’s right foot was placed on a line drawn on the ground 100cm from the Kinect, as described above. The items (talar head palpation being excluded) of the modified FPI described above were then acquired in three different views of the foot whilst the participant stood as still as possible (see Figure 2). Firstly, the participant was asked to stand with the lateral and then the posterior aspects of their right foot facing the Kinect. In each of these first two positions, the participant was in double limb support and instructed to have their feet shoulder width apart in a comfortable position. For the third and final position, the participant had the medial aspect of their right foot facing the Kinect. To allow the camera to see the medial aspect of their foot, the participant’s left foot was placed in a comfortable position behind them and they were instructed to keep their right shank perpendicular to the ground and to have approximately 50% of their body mass through their right foot. The lateral position trial was used to determine the lateral malleolar curvature (FPI item 2) whilst the rearfoot position trial was used to calculate the calcaneal inversion/eversion (FPI item 3). The medial trial was used to establish the ongruence of the medial longitudinal arch (FPI item 5) as well as the talo-navicular joint bulging (FPI item 4). Lastly, to determine the forefoot abduction/adduction (FPI item 6) a combination of the lateral and medial position trials was used. These processes are outlined in greater detail below.

**
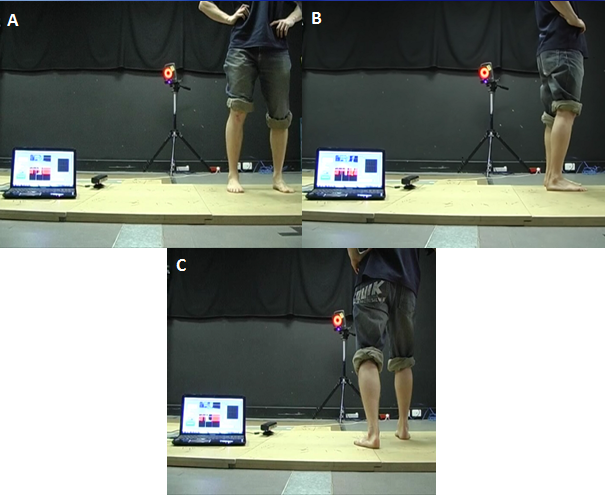
Figure 2. Three positions of the right foot for Kinect assessment of foot posture.** A: Lateral view of the right foot. B: Posterior view of the right foot. C: Medial view of the right foot.

In each of the three positions, the Kinect was used to record two seconds of data. A customised software program was then used to calculate features of lower limb foot alignment and posture using the image and depth sensor data as outlined in the following section. This process was completed on each of the two sessions, approximately one week apart.

**Data Analysis**

Custom made LabVIEW 2009 software (National Instruments, U.S.A.) was used to collect and analyse the data from the Kinect using the Microsoft Software Development Kit (SDK) Beta 2 (Microsoft, U.S.A.). Data were sampled at the native frequency of the Kinect, which is irregular at ≈30Hz. The depth image was converted to the same resolution as the video image using interpolation, and the two images were aligned using a cross correlation function. The real world coordinates of the pixels in the video and depth images were determined using the calibration data extracted from the SDK. When anatomical landmarks or specific positions needed to be identified these were located using the video image, and the corresponding point on the depth map was extracted. For all values the median of five consecutive frames of Kinect data were used.

The lateral position trial was used to determine the lateral malleolar curvature (FPI item 2). Similar to the Vicon analysis, the proximal and distal groove size was calculated and the proximal groove was subtracted from the distal groove, which gave a negative number for a more pronated foot posture. In contrast to the Vicon analysis, to remove noise and improve the accuracy of the depth data, a five point median filter with the centre positioned on the line drawn on the leg was applied to each vertical pixel row. An example of this analysis is provided in Figure 3.

**
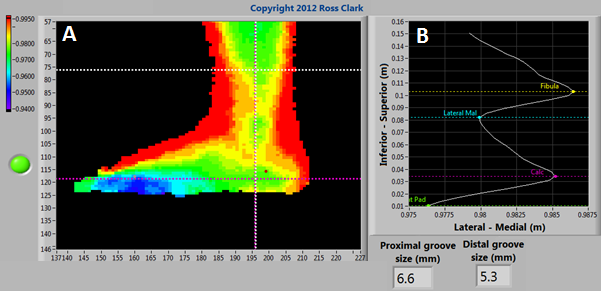
Figure 3. Kinect analysis of the lateral malleolar curvature (FPI item 2).** A: Representation of the depth data, with dark blue being closest to the sensor and red being furthest away. B: Representation of the malleolar curvature recorded from the depth image, with the lower shank, lateral malleolus, lateral calcaneus and fat pad identified. Note: This is a mirror image of the right foot.

For calcaneal inversion/eversion (FPI item 3) the rearfoot position trial was used. The most posterior points of the calcaneus in each pixel row were determined from the depth sensor data and the resultant angle of these points was calculated against the vertical. For the Kinect analysis, a large negative angle indicated a pronated position whereas a large positive angle was indicative of a supinated position. An example of this is provided in Figure 4.

**
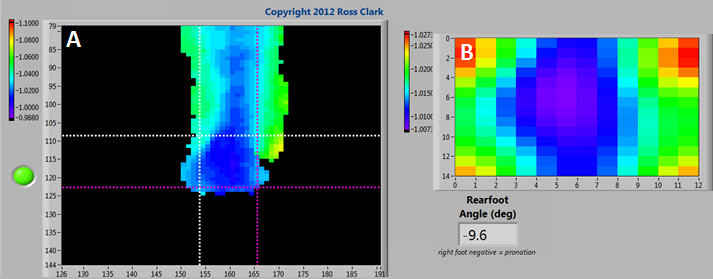
Figure 4. Kinect analysis of calcaneal inversion/eversion (FPI item 3).** A: Representation of the depth data, with dark blue being closest to the sensor and red being furthest away B: Depth data extraction between the cursors to focus on the calcaneus. Note: This is a mirror image of the right foot therefore a negative angle indicates the eversion seen in a pronated foot.

Talo-navicular joint bulging (FPI item 4) was determined from the medial trial by calculating the medial-lateral displacement of the navicular tuberosity in relation to the medial calcaneus. This was done by identifying these landmarks by manually placing cursors on the video image and using the corresponding points on the depth map. Similar to Vicon analysis, a more pronated position was indicated by a more medial protrusion of the navicular tuberosity compared to the medial calcaneus.

The medial trial was also used to determine the congruence of the medial longitudinal arch (FPI item 5) in a similar way to the Vicon analysis. The vertical position of the point in each vertical column of the depth map pixels was identified and plotted along its anterior-posterior position on a 2D scatter plot. The peak vertical position was deemed arch height, and the position on the anterior-posterior axis was calculated relative to the distance between the calcaneus and head of the first metatarsal and expressed as a percentage from the calcaneus. Therefore, FPI item 5 was compared to two measures, arch height and arch peak, derived from the Kinect system, which gave a total of six outcome measures from the Kinect measure of static foot posture. A more pronated position was one with a lower arch height and an anteriorly located peak indicated by a higher percentage. An example of this is provided in Figure 5.


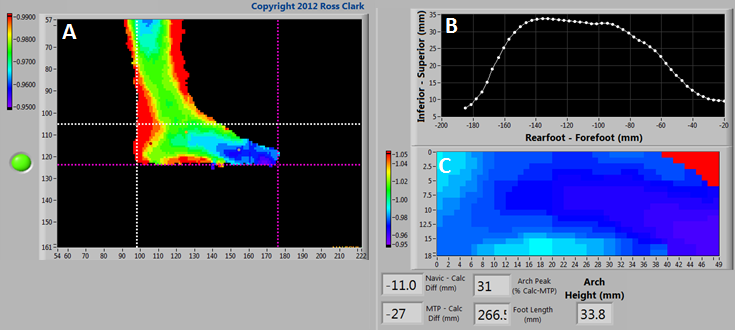


**Figure 5. Kinect analysis of both the medial arch height and arch peak (FPI item 5).** A: Representation of the depth data, with dark blue being closest to the sensor and red being furthest away. B: Representation of the medial arch. C: Depth data extraction between the medial calcaneus and the head of the first metatarsal to focus on the medial arch. Note: This is a mirror image of the right foot.

Lastly, forefoot abduction/adduction (FPI item 6) used both the lateral and medial position trials. The medial-lateral displacement between the lateral calcaneus and the head of the fifth metatarsal was compared with the medial-lateral displacement between the medial calcaneus and the head of the first metatarsal. Similar to the Vicon analysis, the medial displacement was subtracted from the lateral displacement giving a higher score for forefoot abduction and a more pronated foot posture.

**References**

1. Khoshelham K, Elberink SO: **Accuracy and resolution of Kinect depth data for indoor mapping applications.** *Sensors* 2012, **12:**1437-1454.

2. Park S, Yu S, Kim J, Kim S, Lee S: **3D hand tracking using Kalman filter in depth space.** *EURASIP J Adv Signal Process* 2012, **36:**1-18.

3. Clark RA, Pua Y-H, Fortin K, Ritchie C, Webster KE, Denehy L, Bryant AL: **Validity of the Microsoft Kinect for assessment of postural control.** *Gait Posture* 2012, **36:**372-377.
